# Identification of Key Factors for Optimized Health Care Services: Protocol for a Multiphase Study of the Dubai Vaccination Campaign

**DOI:** 10.2196/42278

**Published:** 2023-04-17

**Authors:** Hayette Faroun, Nabil Zary, Khalifa Baqer, Farida Alkhaja, Kareem Gad, Mohamad Alameddine, Hanan Al Suwaidi

**Affiliations:** 1 Institute for Excellence in Health Professions Education Mohammed Bin Rashid University of Medicine and Health Sciences Dubai United Arab Emirates; 2 Dubai Academic Health Corporation Dubai United Arab Emirates; 3 Smart Services Mohammed Bin Rashid University of Medicine and Health Sciences Dubai United Arab Emirates; 4 College of Health Sciences University of Sharjah Sharjah United Arab Emirates; 5 College of Medicine Mohammed Bin Rashid University of Medicine and Health Sciences Dubai United Arab Emirates

**Keywords:** COVID-19, mass vaccination center, MVC, health care services, key success factors, critical success factors, CSFs, service-oriented architecture, SOA, campaign, vaccine, immunize, immunization, inoculation, health information system, HIS, information system, semistructured interviews, Q-methodology, vaccination, health promotion, vaccine campaign, health information, global health, health care service, simulation modeling

## Abstract

**Background:**

Mass vaccination of the global population against the novel COVID-19 outbreak posed multiple challenges, including effectively administering millions of doses in a short period of time while ensuring public safety and accessibility. The government of Dubai launched a mass campaign in December 2020 to vaccinate all its citizens and residents, targeting the population aged >18 years against COVID-19. The vaccination campaign involved a transformation of multiple commercial spaces into mass vaccination centers across the city of Dubai, the largest of which was the Dubai One Central (DOC) vaccination center. It was operational between January 17, 2021, and 27 January 27, 2022.

**Objective:**

The multiphase research study aims to empirically explore the opinions of multiple health care stakeholders, elicit the key success factors that can influence the effective delivery of emergency health care services such as a COVID-19 mass vaccination center, and explore how these factors relate to one another.

**Methods:**

To understand more about the operations of the DOC vaccination center, the study follows a multiphase design divided into 2 phases. The study is being conducted by the Institute for Excellence in Health Professions Education at Mohammed Bin Rashid University of Medicine and Health Sciences between December 2021 and January 2023. To elicit the key success factors that contributed to the vaccination campaign administered at DOC, the research team conducted 30 semistructured interviews (SSIs) with a sample of staff and volunteers who worked at the DOC vaccination center. Stratified random sampling was used to select the participants, and the interview cohort included representatives from the management team, team leaders, the administration and registration team, vaccinators, and volunteers. A total of 103 people were invited to take part in the research study, and 30 agreed to participate in the SSIs. To validate the participation of various stakeholders, phase 2 will analytically investigate one’s subjectivity through Q-methodology and empirically investigate the opinions obtained from the research participants during phase 1.

**Results:**

As of July 2022, 30 SSIs were conducted with the research participants.

**Conclusions:**

The study will provide a comprehensive 2-phase approach to obtaining the key success factors that can influence the delivery of high-quality health care services such as emergency services launched during a global pandemic. The study’s findings will be translated into key factors that could support designing future health care services utilizing evidence-based practice. In line with future plans, a study will use data, collected through the DOC vaccination center, to develop a simulation model outlining the process of the customer journey and center workflow.

**International Registered Report Identifier (IRRID):**

DERR1-10.2196/42278

## Introduction

### Improving Health Care Services

Health care providers are continuously looking to innovate the design and delivery of health care services as it is essential to provide the highest level of patient and public health care [1]. In general, health care systems that can also include the design of health care services are deemed complicated since multiple parties are typically involved, such as health care experts, patients, indirect caregivers, governmental entities, political representatives, and the community [2]. The relationships between these diverse stakeholder groups may impact the planning and delivery of high-quality patient care [2]. Furthermore, Mosadeghrad [3] identified 5 specific features that are needed to provide high-quality health care, including environment, empathy, efficiency, effectiveness, and efficacy.

Gaining insight into the nature of the stakeholder’s interactions and individual perspectives is valuable to consistently enhance the quality of care, ensuring that these 5 topics are also incorporated within health care design [2,3]. These insights can be integrated into a process known as design thinking, which is described as a systematic approach to executing progressive ideas through understanding the patients’ and stakeholders’ needs [1]. Studies have demonstrated the benefit of applying design thinking to the health care industry to meet patient needs and provide new services covering areas such as digital innovation, medical devices, new patient experiences, and upgraded hospital and clinic environments [1].

### Critical Success Factors in Health Care

Another aspect of improving health care services is applying service-oriented architecture (SOA), which was found to reduce costs, improve patient care quality, and incorporate the organization’s legacy system with updated IT infrastructure to be better aligned with the organization’s principles and targets [4]. In addition, SOA encompasses the concept of reusability and focuses on streamlining processes within an integrated health care system [4].

Research surrounding the implementation of SOA in health care has identified critical success factors (CSFs) that can influence this process and are considered valuable for its success. Koumaditis et al [4] developed a proposed CSFs model comprising of 21 factors that can be used as a framework for the successful implementation of SOA in health care and the design of effective services. The factors were categorized into 5 groups consisting of managerial, operational, strategic, IT infrastructure, and organizational topics. The theory of proposing the CSFs model can support health care practitioners and researchers in classifying the importance of each CSF and its impact within each category. The significance of creating an integrated health information system is noted to reduce medical errors, improve medical data, and enhance governance and decision-making [4].

### Health Care Services in Emergency Situations

During the COVID-19 pandemic, one of the health care services that needed to be immediately deployed was the creation of emergency vaccination centers to support the vaccination strategy [5,6]. When discussing the feasibility of introducing vaccine centers, multiple factors are considered, including the structure of the local health care system, infrastructural strength, and monitoring the success of the vaccination campaign [7]. Mass vaccination of the global population posed multiple challenges, including the effective administration of millions of doses in a short period of time while ensuring it can be safely and widely accessible to everyone [8].

The typical method to improve the effectiveness of mass vaccination during health emergencies or a global pandemic is the development of emergency points, also known as points of dispensing (PODs) [6]. PODs are physical locations that provide medical services such as vaccines to large populations in a short time frame. They differ from typical medical facilities as their primary purpose is to fairly provide medical supplies on a large scale [6]. Mass vaccination centers (MVCs) or PODs are commonly placed in nontraditional spaces or short-term sites such as converting parking lots or larger indoor areas due to the exceptional circumstances of the situation [8].

### COVID-19 Management in Dubai

The novel COVID-19 outbreak was first declared a global pandemic by the World Health Organization on March 11, 2020 [9]. Due to the virus’s fast-spreading nature, the pandemic began to globally disrupt daily life and compelled countries to announce extended periods of nationwide lockdowns and impose travel restrictions to curb the spread of the virus [10]. The United Arab Emirates (UAE) was no exception and began a partial lockdown campaign on March 22, 2020 [11]. Regaining any level of normalcy hinged on understanding a suitable way to manage symptoms from COVID-19 infection, the discovery of a possible treatment, and more importantly, the administration of effective vaccines [8,9]. The second half of 2020 carried promising news with the discovery and the initiation of mass production of several vaccines, initiated by the World Health Organization as part of their global effort to manage the pandemic [8,9]. Looking more closely at the UAE, the response protocols set by the National Emergency, Crisis, and Disaster Management Authority included large-scale testing, monitoring outbreak cases, creating emergency field hospitals, global cooperation, and the deployment of vaccination campaigns [12].

The government of Dubai, for example, launched a mass campaign to vaccinate all its citizens and residents who met the criteria in December 2020, also targeting the population aged >18 years. The collaborating authorities managing the launch of this campaign involved the Dubai Health Authority and Dubai’s COVID-19 Command and Control Center. The expertise throughout the collective teams complemented each other; the Dubai Health Authority placed a medical guideline for the administration of the vaccines and shared updated protocols, whereas Dubai’s COVID-19 Command and Control Center developed a strategy to open and manage multiple vaccination centers across the city. The implementation of this program stretched the resources of various governmental agencies to swiftly achieve and create accessible public vaccination PODs. It included transforming commercial spaces into dedicated vaccination centers, the largest of which was the Dubai One Central (DOC) vaccination center. This was the first vaccination center opened in Dubai and was operational between January 17, 2021, and January 27, 2022.

### DOC Vaccination Center

**Figure 1:**
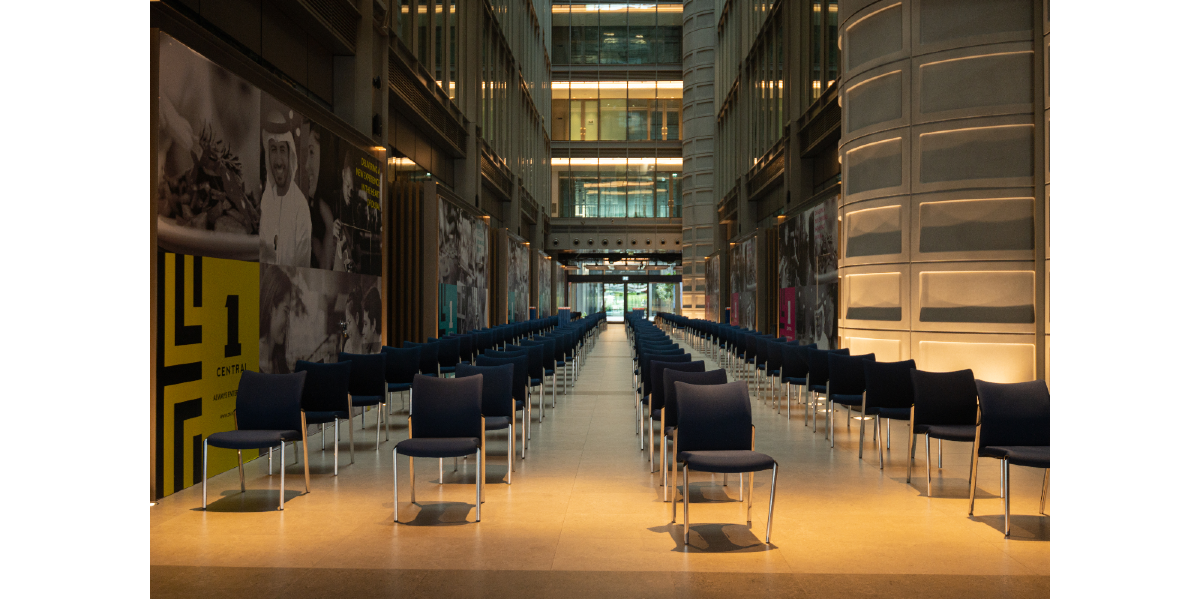
Preregistration waiting area of the vaccination center in Dubai One Central.

The empty commercial spaces at DOC were swiftly transformed and equipped to become a fully functioning vaccination center in just 72 hours. Figure 1 shows one of the waiting halls in DOC. The transformation involved multiple streams of action, such as the placement of all necessary medical equipment, planning for the ultra-cold supply chain of vaccine vials and their storage, recruiting the required staff, as well as the mapping out of the customer journey and flow of people in and out of the center.

As a result, the team at the center administered 1,476,776 Pfizer-BioNTech vaccine doses (complete appointments) against the infection of COVID-19 in one year (367 operational days). They are considered as complete appointments once the customer completes the entire process of registering for and receiving a COVID-19 vaccine dose, whether it is the first, second, or third dose. The DOC vaccination center received between 147,836 and 211,859 customers per month during the peak operational times (May 2021 to September 2021), vaccinating between an average of 5280 and 7062 customers per day. Notably, the original capacity of the center was designed to hold a total of 4000 customers per day; however, through various operational improvements, the center was able to receive and vaccinate over 10,000 people per day.

### Aim and Research Questions

Our review of the literature identified multiple studies that discussed the importance of exploring the patient’s perspective when designing an optimized model to deliver a more efficient health care service [1,13,14]. To ensure that we are considering a more comprehensive perspective, our study aims to empirically explore the opinion of multiple health care stakeholders and elicit the key success factors influencing the delivery of quality care at the DOC vaccination center.

Through a comprehensive research design, the following 3 research questions will be addressed:

1. What key success factors can influence the delivery of a health care service during a public health emergency, such as a vaccination center?
2. How do these CSFs relate to one another?
3. To what extent do these CSFs impact the delivery of health care services?

## Methods

### Study Design

**Figure 2:**
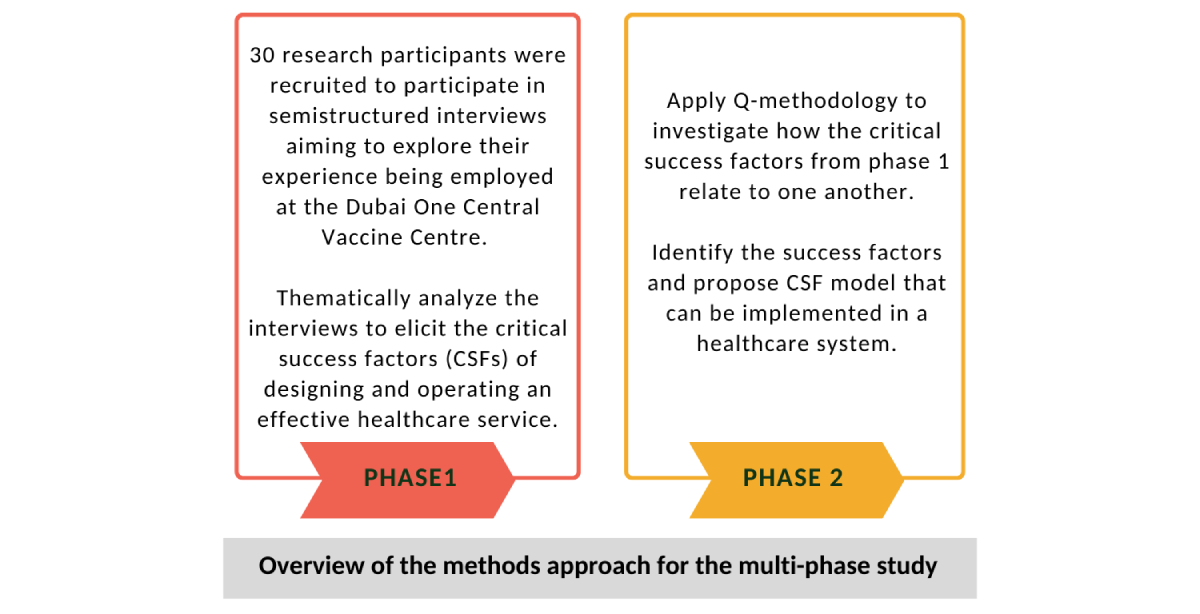
Overview of the methods approach for the multiphase study.

The study is being conducted by the Institute for Excellence in Health Professions Education at Mohammed Bin Rashid University of Medicine and Health Sciences, located in Dubai, UAE, between December 2021 and January 2023. To better understand the CSFs of the DOC vaccination campaign, this study follows a multiphase design divided into 2 phases, as illustrated in Figure 2. This 2-step approach aims to investigate the key success factors that supported the design and delivery of a health care service implemented as part of nationwide efforts to combat a global pandemic. It further aims at documenting the efforts and exploring the operations of the first COVID-19 vaccination center opened in Dubai, the DOC vaccination center.

### Phase 1: Conducting Semistructured Interviews

A qualitative design was applied to fulfill the study’s aim and answer the first research question. Individual semistructured interviews (SSIs) were conducted via Microsoft Teams with a sample of staff and volunteers (n=30) working at the DOC vaccination center. The staff and volunteers of DOC were considered key contributors to the design and execution of the vaccination program. A total of 103 people were invited to participate in the research study, and 30 people agreed to participate in the SSIs.

Stratified random sampling was used to select the participants. The interview cohort included representatives from team leaders, the management team, the administration and registration team, vaccinators, and volunteers. The composition of the participants’ roles is included in Multimedia Appendix 1. The inclusion criteria to participate in the study’s interview phase included participants aged 18-70 years, who could fluently communicate in Arabic or English, and who worked for a minimum of 3 months at the DOC vaccination center. Based on previous research, a target of at least 30 participants is needed for the sample size to reach thematic saturation and were selected based on their experience at DOC and criteria eligibility [15].

The study’s cohort was recruited via email or phone invitation. Before the start of any interview, the research participants were asked to sign a consent form that outlined their rights as a research participant and complete a questionnaire that collected general sociodemographic data before the start of the interview, which can be found in Multimedia Appendices 2 and 3. The approximate duration of the interviews was scheduled for 60 minutes.

The interview protocol was developed to capture the study’s objectives and included 2 main elements: the opening statement by the interviewer and the interview script, including a set of questions acting as a guide during the discussion (Multimedia Appendix 4). The opening statement introduces the researcher, the interview’s intention, how the interview result will be utilized, consent to the interview, confidentiality of the interviewee’s identity, and options to withdraw at any stage of the process [16]. Before finalizing both versions of the interview guide, a pilot round of SSIs was performed with a sample participant group (n=8). It comprised experts who led the same roles as outlined in Multimedia Appendix 1. The process led to the interview protocol being revised twice after the pilot phase. In addition to testing the interview guide, if the opening statement and flow of questions were understood, the researcher practiced probing to extract key insights and expand on certain topics where needed. The interview guide was then translated to Arabic (Multimedia Appendix 5) to accommodate the language preferences of almost half (14/30, 47%) of the participants. The Arabic language SSI guide was revised by an independent reviewer and later back-translated by another translator to ensure it matched the copy of the English SSI guide.

Finally, the interviews were recorded through Microsoft Teams for documentation purposes, which later assisted with the transcription process. The SSIs conducted in English (n=16) were outsourced to be transcribed using the verbatim method and then revised by a researcher on the project. The Arabic interviews were also transcribed verbatim, translated to English, and then independently revised by a researcher. Finally, the content is currently in the process of being thematically analyzed using MAXQDA 2020 (VERBI Software) [17]. The thought process behind the thematic analysis is to use the topics outlined in the interview guide (Multimedia Appendix 4) as the main themes. During the coding process, subthemes are intended to emerge, which will shape the list of key success factors considered as the driving force behind operations of the vaccine center. Each of the themes and subthemes were assigned a number, and any mention of a specific theme (success factor) across all 30 interviews will be tabulated and categorized, as viewed by the research participants. The thematic analysis will be conducted by a researcher and reviewed by 2 independent reviewers. If discrepancies are found among the answers, they will be referred to the lead investigator for a final decision.

### Phase 2: Applying Q-Methodology

**Figure 3:**
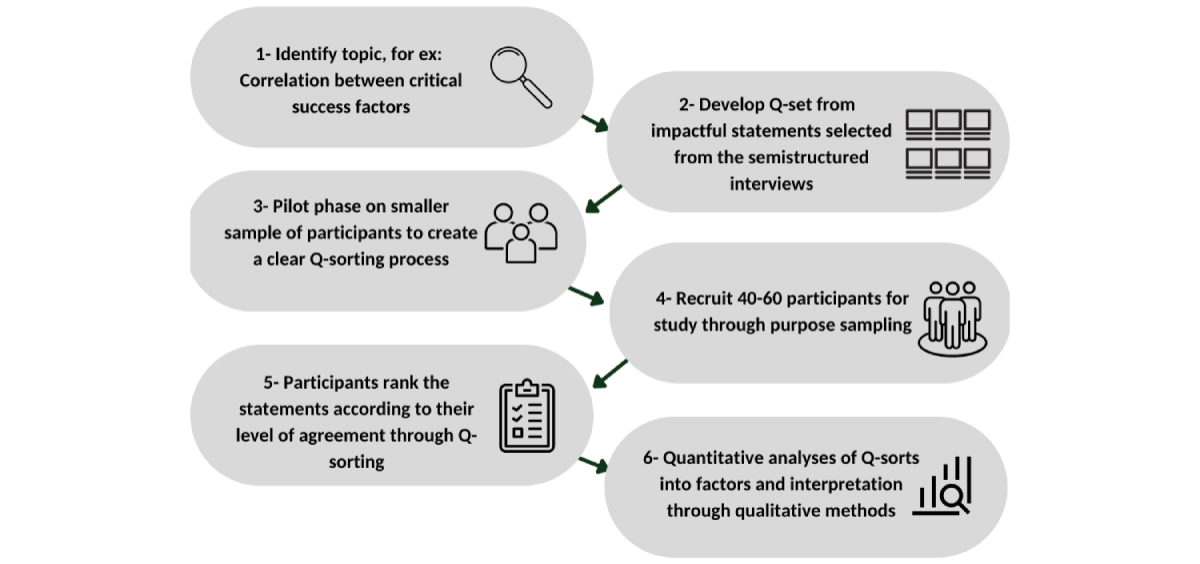
Stages of the Q-Methodology to study the participants' perceptions collected from phase 1. Image inspired by the stages of Q-methodology from Churruca et al [2] which is published under Creative Commons Attribution 4.0 International License [18].

Combining qualitative and quantitative techniques, the Q-methodology aims to investigate one’s subjectivity [1] analytically and will be applied to empirically investigate the opinions obtained from the research participants during phase 1. The entire process of the Q-methodology is illustrated in Figure 3 and is summarized across 6 stages. In addition, this technique has illustrated that it is suitable for use when various health care stakeholders are involved (Multimedia Appendix 1) [1].

To answer the second research question, a topic will be identified for the Q-methodology to examine how the identified success factors from phase 1 relate to one another. The “Q-set” will be developed based on the most common and impactful answers selected from the thematic analysis of the SSIs. This “Q-set” will be shared with a smaller sample group (n=10), and they will be asked to rank each of the statements based on purposefully defined criteria such as perceived importance [1]. After gathering feedback from the participants in the pilot phase, the content will be modified if needed and shared with a larger cohort (n=40 to 60) [1]. The inclusion criteria to participate in this phase of the study includes participants aged 18-70 years, who can fluently communicate in English or Arabic, and who worked for a minimum of 3 months at any of the vaccination centers in Dubai.

The next step is data collection and will include “Q-sorting,” where each research participant ranks the statements in the “Q-set,” following the same scale set in the pilot phase. After purpose sampling, a collection of the ranked statement by an individual, known as the “Q-sort,” will be statistically analyzed using the inverted factor analysis methods [1]. During the “Q-sorting” stage, individuals will be asked postsorting questions about their experience participating in the research and their knowledge of the topic at hand, which could lead to additional qualitative insights [1].

Finally, the CSFs that may impact the delivery of health care services will be identified after discovering the relation between the factors. These CSFs will be mapped and categorized following the proposed model by Koumaditis et al [4].

### Ethics Approval

The Dubai Scientific Research Ethics Committee approved the study in November 2021 (DSREC-11/2021_04).

### Informed Consent and Data Security

The research population included members directly involved in developing and implementing the COVID-19 vaccination campaign in Dubai and at the DOC vaccination center. All participants signed a consent form before the interview and were notified of the study’s purpose in written and verbal communication. The identity of all participants will remain anonymous during the submission of the results for phases 1 and 2 of the study. All study data will be kept on a secure, password-protected computer accessible only to the research team (principal investigator and research assistant).

## Results

To fulfill the first research question, as of July 2022, 30 SSIs were conducted with the research participants outlined in Multimedia Appendix 1. On average, each interview lasted 45 minutes and ranged between 25 and 84 minutes.

## Discussion

### Expected Findings

The results from the project’s first phase are expected to elicit the critical success factors, enablers, and barriers of the design and operation of the COVID-19 vaccination center at DOC.

Results from the project’s second phase will highlight how the success factors identified in phase 1 will relate to each other, answering the study’s second research question. Additionally, the expected results from the analysis will feature patterns of similarities and differences from the Q-sets. Finally, and to answer the third research question, we will identify the extent of these success factors’ impact on the delivery of health care services.

### Strengths of the Study

The SSIs were conducted to explore people’s perceptions about the DOC vaccination center and elicit the key success factors surrounding its design and implementation. Another strength of the research study is the number and quality of interviews completed. The researchers organized 30 interviews—the recommended number of SSIs that need to be completed to provide meaningful results for a research study [15]. The group of interviewees included employees and volunteers from diverse professional backgrounds. The varying roles each of the interviewees held at the vaccination center (refer to Multimedia Appendix 1) provided the study with richer and diverse insights [19]. Furthermore, the open-ended question format of the SSIs allowed the participants to focus on each topic in detail and share varied responses based on their individual experiences [20]. This approach helps reduce the risk of bias relating to the researcher’s preconceptions about the themes, as this style of data collection allows the use of follow-up probes throughout the discussion as directed by the participant [20].

In addition to completing the SSIs, the Q-methodology will focus on allowing participants to express their opinions about the subject while reducing former expectations and possible biases introduced by the researcher. It is also known as a people-centric approach that can provide a deeper view on the matter and is beneficial when studying complex health care topics [1]. This method is not commonly found in health care research and can potentially be a valuable tool [1].

The result of our research can help bridge a noted gap in the literature [4] as we explore each of the CSFs independently, their relation to each other and the health care service, as well as develop a CSFs model that can be applied to any level within a health care organization or service to improve the quality of patient care and organization of data [4]. Combined with the implementation of SOA, there is a real opportunity to achieve interoperability and flexibility to update medical processes in line with new regulations and improve decision-making [4]. According to research in this area, developing SOA culture and governance from the leadership are seen as a driving force for resisting change and successful execution [4].

It is noted that there is limited information in the medical literature on the systematic process of creating MVCs. Although mass vaccination campaigns have been mentioned as a general component for managing communicable diseases since the 1970s during the spread of H1N1 influenza, there is a lack of clear criteria to defining an MVC, such as pertaining to the number of doses and/or number of people served in a day [8]. For example, in the spring of 2021, Euro Disney Paris was transformed into an MVC with the capability of serving 1000 doses/day, whereas in Italian cities, they were able to vaccinate over 4000 people in one day [8]. Global health experts are executing their own strategies and procedures to administer the COVID-19 vaccines as the concept has more prominently emerged within the last 2 and a half years [8].

### Comparison to Other Works

Studies surrounding the development of MVCs specific to the COVID-19 pandemic are still considerably low [8,21]. They are typically found to focus on documenting the MVC process, analyzing the vaccination system, and understanding people’s perceptions about the COVID-19 vaccine [20,22]. However, the outcome from these studies is not usually connected to the enhancement of more general health care services and improving patient care. For example, Brambilla et al [21] studied the process surrounding mass vaccination. They outlined the method of developing a scalable and replicable MVC model in Lombardy, Italy, which can be adapted and created in non–health care locations, whereas Andrade et al [22] focused their research on investigating the UAE’s population’s motivation to receive the vaccine and its correlation to their level of understanding, acceptance, and conspiracy beliefs related to the vaccine. Another example of a recently published study illustrated the application of Lean tools to develop an MVC and investigated its effectiveness and whether this approach increases the efficiency of mass vaccination sites [23].

Since MVCs for COVID-19 are believed to be the first mass vaccination effort in the present era [8], it is considered integral to document the process of creating a high-volume MVC such as the DOC vaccination center and the collaborative efforts to safely administer over 1.4 million doses in the span of one year to everyone that required it. In addition to documenting these efforts, this study also aims to connect the results from understanding the key success factors of implementing an MVC to its potential impact on the delivery of health care services and improving patient care.

Knowledge generated from this study conducted in the Gulf Cooperation Council and the Middle East region will contribute to a more accurate global representation of the success factors, as we aim to empirically explore the opinion of multiple health care stakeholders and elicit the key success factors influencing the delivery of a health care service during a public health emergency, such as a vaccination center.

### Limitations of the Study

Due to restrictions imposed because of the COVID-19 pandemic, the SSIs were completed remotely, via the videoconferencing platform Microsoft Teams. This was often considered a challenge as there were technical difficulties while the interviews were taking place such as unreliable internet connection. Utilizing an online platform to collect data may have impacted the quality of recordings, which in turn may have affected the quality of the transcriptions. Additionally, attempting to schedule at least 30 interviews in a short time frame before the center’s closure proved difficult to plan around the employee’s schedules, and the interview schedule was continuously delayed. The SSIs were conducted with a sample of DOC vaccination center’s staff and volunteers before the closure of the center by 2 months and were completed shortly afterward. Therefore, the time between data collection and the closure of the DOC vaccination center may have contributed to recall bias as the interviewees were asked to recollect events from the past. Finally, due to the workload of the front-line workers at the DOC vaccination center, pandemic fatigue may have also been a factor that could have influenced their responses in the interviews.

### Future Work

Future research will leverage the data collected to contribute to the medical informatics field, specifically with the aims to explore approaches that can effectively analyze big data, include predictive analytics, and optimize decision-making [24]. In line with the future plans, a study will use data, collected through the DOC vaccination center, to develop a simulation model outlining the process of the customer journey and center workflow.

The simulation model will focus on capturing the data at 3 significant milestones, including when the center reached a daily capacity of administering 1000 doses, 4000 doses, and 10,000 doses to customers. This will showcase the center during various operational phases and the improvements that took place during each stage and identify areas that needed more enhancements to increase operational efficiency. Simulation modeling has been identified as a smart tool that offers the prospect of testing various scenarios using a cost-effective approach, obtaining solutions for obstacles during certain health care service phases, comprehensively visualizing the process [8].

This analytical phase of the study will focus on the operational aspect of designing a health care service and how it can assist with the decision-making process through an optimized health care service model [24]. In a way, the pandemic accelerated the growth of designing and delivering a high-capacity vaccination center. Therefore, it is recommended that the next phase of planning for health care services is to focus on operational efficiency, its link to patient satisfaction, high-quality care, and the need to apply a standard operating procedure across various processes and facilities [4].

### Conclusion

The study provides a comprehensive 2-phase approach to eliciting the CSFs that can influence the delivery of high-quality health care services such as emergency services launched during a global pandemic. The findings from the study will be translated into key factors that could be optimized and applied when designing future health care services, utilizing evidence-based practice. Additionally, the results from the study conducted in the Middle East region will contribute to a more globally accurate representation of CSFs. Finally, the future work from this research study will explore methods to analyze data collected from the DOC vaccination center, include predictive analytics, and optimize decision-making.

## Appendix

Multimedia Appendix 1 Table describing each of the research participants’ roles at the Dubai One Central vaccination center and number of participants from each group.

Multimedia Appendix 2 Consent form shared with all participants prior to the start of their semistructured interview.

Multimedia Appendix 3 Questionnaire which was used to collect sociodemographic information about each of the participants before their interview.

Multimedia Appendix 4 Final version of the interview guide in English, developed for the semistructured interviews. The table outlines the themes of the groups of questions, their number, scheduled questions, and follow-up probes that could be used throughout the discussion.

Multimedia Appendix 5 Final version of the translated interview guide in Arabic was developed for the semistructured interviews. The table outlines the themes of the groups of questions, their number, scheduled questions, and follow-up probes that could be used throughout the discussion.

## Abbreviations

CSF: critical success factor
DOC: Dubai One Central
MVC: mass vaccination center
POD: point of dispensing
SOA: service-oriented architecture
SSI: semistructured interview
UAE: United Arab Emirates

## Data Availability

The data sets generated during and/or analyzed during the current study are available from the corresponding author on reasonable request.
