# Supplementary material for: Identification of Key Factors for Optimized Health Care Services: Protocol for a Multiphase Study of the Dubai Vaccination Campaign
Source: JMIR Res Protoc. 2023 Apr 17;12:e42278. doi: 10.2196/42278 (PMC10131770; doi:10.2196/42278)
Supplement: Multimedia Appendix 1 [file resprot_v12i1e42278_app1.pdf]

**Role of Employees at Dubai One Central Vaccination Center**	**Number of Participants**
Leadership	2
Management	4
Administration, Registration and Data Entry Operators	7
Vaccinators	12
Volunteers	5
**Total number of participants**	**30**
