# Supplementary material for: Identification of Key Factors for Optimized Health Care Services: Protocol for a Multiphase Study of the Dubai Vaccination Campaign
Source: JMIR Res Protoc. 2023 Apr 17;12:e42278. doi: 10.2196/42278 (PMC10131770; doi:10.2196/42278)
Supplement: Multimedia Appendix 2 [file resprot_v12i1e42278_app2.pdf]

Investigating the Implementation of the COVID-19 Vaccination Program in Dubai: The Case of Dubai One Central

Mixed-Methods Research Study

Consent Form for Semi-Structured Interviews

I [**insert full name here**], voluntarily agree to participate in this research study.

I understand that even if I agree to participate now, I can withdraw at any time or refuse to answer any question without any consequence of any kind.

I have had the purpose and nature of the study explained to me verbally and in writing, and I have had the opportunity to ask questions about the study.

I understand that participation involves answering questions on the research topic.

I understand that there will be no fees for participation.

I understand that I will not directly benefit from participating in this research.

I agree to my interview being audio and visually recorded.

I understand that all information I provide for this study will be treated confidentially.

I understand that in any report on the results of this research, my identity will remain anonymous.

This will be done by deidentifying any material related to my interview or the identity of people I speak about.

--- ------------------------

Signature of Participant Date

--- ------------------------

Signature of Researcher Date
