# Supplementary material for: Identification of Key Factors for Optimized Health Care Services: Protocol for a Multiphase Study of the Dubai Vaccination Campaign
Source: JMIR Res Protoc. 2023 Apr 17;12:e42278. doi: 10.2196/42278 (PMC10131770; doi:10.2196/42278)
Supplement: Multimedia Appendix 3 [file resprot_v12i1e42278_app3.pdf]

Investigating the Implementation of the COVID-19 Vaccination Program in Dubai: The Case of Dubai One Central

Questionnaire for Participants of the Interview

Interview Date (DD/MM/YYYY): ________________

1. What is your gender?
   a. Female
   b. Male

2. How old are you?
   a. 18 - 29 years old
   b. 30 - 39 years old
   c. 40 - 49 years old
   d. 50 - 59 years old
   e. 60 and older

3. Are you temporary or permanent DHA staff?

   ____

4. Do you have experience working in a professional healthcare setting?

   ____

4.a. If yes, please indicate how many years of experience you have, and **mention your** profession below.

a. 0 - 3 years
b. 4 - 6 years
c. 7 - 9 years
d. 10 - 12 years

e. 13 years or more

____

4.b. If no, can you please tell us more about the industry you professionally worked in, and your past/ current job?

____

5. How long did you work at the Dubai One Central vaccine centre?
   a. 1 - 3 months
   b. 4 - 6 months
   c. 7 - 9 months
   d. 10 - 12 months
   e. Over 1 year

6. Did you work at other COVID-19 vaccination centres? If yes, kindly indicate the name of the name/s of the centre.
   a. Yes
   b. No

____
