# Supplementary material for: Identification of Key Factors for Optimized Health Care Services: Protocol for a Multiphase Study of the Dubai Vaccination Campaign
Source: JMIR Res Protoc. 2023 Apr 17;12:e42278. doi: 10.2196/42278 (PMC10131770; doi:10.2196/42278)
Supplement: Multimedia Appendix 4 [file resprot_v12i1e42278_app4.pdf]

Theme	Item Number	Scheduled Question Stems and Probes
**Dubai government's response to curb the COVID-19 pandemic**	1	**1- Can you tell me the reason the vaccine center was created at Dubai One Central (DOC)?**
**Workflow mapping of Dubai One Central**	2	**2- What is/ was your assigned job (task) at DOC?**<br>**Probes:**<br>• Can you describe your role in the implementation of the vaccination program at DOC?<br>• Which phase or phases are you involved in during the vaccine campaign?
**Transformation mapping of Dubai One Central**	3	**3- What was unique about the center setup at DOC?**<br>**Probes**:<br>• How was DOC chosen as the location for the creation of the vaccination center?<br>• Was there a vision in mind for the space when the transformation started?<br>• How did you help with the transformation of the space to be a functioning vaccine center?
**Barriers to program implementation**	4	**4- Did you and your team face any challenges at the vaccine center?**<br>**Probes**:<br>• How did you overcome these challenges?<br>• How did you deal with vaccine hesitancy amongst customers?
**Enablers to program implementation**	5	**5- What resources were available to you and your team at the center?**<br>**Probes**:<br>• How did these resources help you create opportunities?<br>• How were you able to accommodate and serve the maximum number of customers possible?

**Necessary improvements completed at Dubai One Central**	6	**6- What improvements happened at DOC from a systems perspective?**<br>**Probes:**<br>• What improvements happened on a personal level?<br>• Can you think of any areas of improvements that could have been implemented at DOC?
**Critical Success Factors (CSFs)**	7	**7- In your opinion, what are the main success factors that supported the vaccination program at DOC?**<br>**Probes:**<br>• How did the support you received from team leads or managers affect your role at the center?<br>• How did that contribute to your role at DOC?
**Program Outcomes: accomplishments**	8	**8- What are the key accomplishments that were achieved at DOC?**<br>**Probes:**<br>• What are the key accomplishments that were achieved on a personal level?
**Program Outcomes: talent building and resource sharing**	9	**9- How do you intend to use your experience from DOC?**
**Program Outcomes: new healthcare standards**	10	**10- Can you tell me about Dubai's healthcare facilities?**<br>**Probes:**<br>• How do you imagine the progress of the healthcare facilities in Dubai in the future?
**Future practice recommendations**	11	**11- Do you have recommendations for future practices?**
		**Are there any other points you would like to add?**
