# Supplementary material for: Identification of Key Factors for Optimized Health Care Services: Protocol for a Multiphase Study of the Dubai Vaccination Campaign
Source: JMIR Res Protoc. 2023 Apr 17;12:e42278. doi: 10.2196/42278 (PMC10131770; doi:10.2196/42278)
Supplement: Multimedia Appendix 5 [file resprot_v12i1e42278_app5.pdf]

المواضيع	رقم الموضوع	الأسئلة المقررة والأسئلة الثانوية المحتملة
**استجابة حكومة دبي للحد من جائحة كوفيد-19**	1	**1- هل يمكنك اخباري عن سبب إنشاء مركز اللقاحات في دبي ون سنترال؟**
**استكشاف خطوات سير العمل في مركز دبي ون سنترال.**	2	**2- ما كانت وظيفتك في المركز؟**<br><br>أرجو وصف دورك في مركز دبي ون سنترال للتطعيم؟<br><br>ما هي المرحلة أو المراحل التي شاركت فيها خلال حملة التطعيم؟
**استكشاف خطوات التحول لمركز دبي ون سنترال.**	3	**برأيك ماذا يميز مركز ون سنترال؟ أو برأيك ما مميزات مركز ون سنترال؟**<br><br>كيف تم اختيار موقع دبي ون سنترال لإنشاء مركز للتطعيم؟<br><br>هل كان يوجد تصميم معين للمساحة عندما بدأ التحول؟<br><br>كيف ساهمت في جعل مركز دبي ون سنترال ليصبح مركز صحي فعال لإعطاء لقاحات للكوفيد-19 ويقدم خدمات بجودة عالية؟
**الحواجز الذين أدوا الى تحديات تنفيذ حملة التطعيم.**	4	**هل واجهت أنت وفريقك أي تحديات في المركز؟**<br><br>كيف تغلبت على هذه التحديات؟<br><br>هل واجهت أي عملاء يترددون في أخذ اللقاح، وكيف تعاملت معهم؟
**عوامل التمكين التي أدت الى تنفيذ حملة التطعيم.**	5	**ما الموارد التي كانت متاحة لكم؟**<br><br>كيف ساهمت هذه الموارد في الاستفادة من الفرص؟<br><br>ما كانت الخطة الذي مكنتكم على استيعاب وخدمة أكبر عدد ممكن من العملاء؟

**التطورات التي حصلت خلال العام الماضي.**	**6**	**ما هي التحسينات التي حدثت من منظور الأنظمة في دبي ون سنترال؟**<br><br>ما هي التحسينات التي عملتها على المستوى الشخصي؟<br><br>برأيك ما هي الإجراءات التي يمكن تحسينها؟
**العوامل الرئيسية (الحاسمة) لضمان نجاح حملة التطعيم الشاملة في دبي.**	**7**	**ما هي عوامل النجاح الأساسية التي دعمت حملة التطعيم في دبي ون سنترال؟**<br><br>كيف أثر الدعم الذي تلقيته من قادة الفريق أو المدراء على دورك في المركز؟
**نتائج الحملة: الإنجازات**	**8**	**ما هي برأيك الإنجازات الرئيسية التي تحققت خلال الحملة؟**<br><br>ما هي إنجازاتك الرئيسية على المستوى الشخصي؟
**نتائج الحملة:**<br>مشاركة الخبرة ونمو **المواهب**	**9**	**كيف تنوي استخدام تجربتك من مركز دبي ون سنترال؟**
**نتائج الحملة: خلق معايير صحية جديدة**	**10**	**ما رأيك في مرافق الرعاية الصحية في امارة دبي؟**
**نصائح لحملات مستقبلية.**	**11**	**هل لديك نصائح لحملات أو برامج مستقبلية؟**
		**هل هناك أي نقاط أو ملاحظات أخرى تود إضافتها؟**
